# The Proximate Causes of Decay of the Teeth of Americans

**Published:** 1878-07

**Authors:** 


AMERICAN JOURNAL

OF

DENTAL SCIENCE.

Vol. XII. THIRD SERIES—JULY, 1878. No. 3.

### ARTICLE I.

### *The Proximate Causes of Decay of the Teeth of Americans.*

BY PROF. HODGKIN.

[Read before the Baltimore Society of Physicians and Surgeons, May 2nd, 1878.]

Mr. President and Gentlemen.—At our last meeting I tried to show you as well as I was able what I conceived to be the remote causes of dental caries, and I trust that the views then presented are sufficiently fresh in your minds to obviate any repetition of them now; and I only refer to the fact that I summed up as the great cause of this malady, imperfect calcification of the teeth consequent upon imperfect nutrition in intra-uterine life.

This theory, while committing us in appearance to the doctrine that "as teeth are formed, so they remain," only in fact commits us to an acceptance of this doctrine so far as the enamel is concerned—the question of nutrition of dentine is one which we need not discuss here. It is sufficient to know that the enamel is imperfectly coalesced and that the open sutures or fissures left from incomplete blending of the points of calcification, leave open roads for the entrance of the active agents in causing decay.

You are aware of the fact that dentine has the property of further calcification, that is, that teeth become harder and denser as age progresses, that the fibrillæ calcify, and that the pulp decreases in size, calcific deposit occuring on its periphery. You are further aware that this is sometimes a physiological manifestation in the presence of pathological conditions—that decay is headed off by what the histologists have called the "zone of consolidation," the unattacked substance of the tooth increasing in density, and forming a more or less perfect barrier to the encroaches of the disease outside of this barrier. But while all this is true of dentine, thus giving ground for the chemico-vital theory of decay, yet it is not at all true of enamel. For if the teachings of our physiologists be true, enamel is calcified from without inwardly—the enamel organ is on the outside of the tooth, and as a matter of course is dissociated with its after eruption.

But this is aside from the question of the "proximate cause of decay," which is what concerns us more immediately. What are the agents which attack the hard vitreous enamel, burrowing into the dentine, penetrating to the pulp chamber, and devastating those beautiful structures, at once the ornaments of beauty and the agents of preliminary digestion ?

We feel the importance of the answer to this question,—all of us,—although it should be that we stand powerless in the presence of this destroyer. To know what is the cause of the trouble is always a step on the road to its prevention. Is it a question of simple chemical action, or do vital forces have their play here ? Is it true that teeth are attacked from without only, or is it true that they are insidiously sapped from within ? We have no time here, nor is it the place to take up these questions in anything like a rigid investigation of their merits. Theories of all sorts have been advanced. The simply chemical one, which finds in an acid condition of the salivary and mucous fluids, or corroding discharges from the gums; or of acids arising from the decomposition of the particles of food retained between and in contact with the teeth; or the return of corrosive elements, as chloride, bromine, &c., after administration of the salts of these elements, and their decomposition in the system, by elimination through the salivary glands. I simply mention these. A strong party finds in the fact that the teeth of pregnant females seem to decay faster than those of others not in that interesting condition, reason for belief that the teeth are robbed from within to supply the lime salts, the wants of the growing fœtus, or at least that they fail to receive nutrition. This theory of course assumes the nutrition of dentine. Another party find in the mysterious *leptrothix buccalis*, a microscopic parasite or fungus found in tooth-substance, a cause of decay, and some have seen, so sharp is human sight when prejudiced, these anamalculæ burrowing their way into the depths of the tooth; and some have even suggested that the pain felt under the sharp excavator of the dentist is due to the disturbance of these creatures and their seeking refuge in the dentinal tubuli.

But enough of these theories, for as the tooth-ache is the most practical of facts, which neither the greatest savant or dreamy visionary can ignore, it behooves us to be practical, and to be practical we must be analytic, and know what are the substances acted upon, and what the forces and agents acting upon those substances. And you will all confess that the pain of a "raging fang" is practical enough, and the chairs and instruments of ours do not smack greatly of philosophy.

> "Of all our pains since man was curst,
> I mean of body, not the mental,
> To name the worst among the worst,
> The dental sure is transcendental."

And although we may make a joke of it, it is the grimest joke that man ever grinned at.

For a moment then let us be analytic and examine the structure of tooth-substance. The enamel is on the surface, is first attacked by caries, and we will take that up, as the most densely calcified structure of the body.

Very briefly, it is composed of long, slender, mostly hexagonal rods, or prisms, their inner ends resting on the dentine, and the outer forming the surface of the tooth. It is composed of "solid, slender fibres of phosphate, carbonate and fluate of lime, the lime-salts constituting 99-100 parts of its substance. The remaining part is animal matter and water. I state this purposely that you may see that tooth-structure, at least so far as enamel is concerned, has no vitality, and as a consequence has no *vital* resistance to decay. The question then narrows down to a single general one—the chemistry of decay. Whatever part vital forces may play in other parts of the body, and whatever influence the repelling forces of nature may bring into requisition elsewhere, here it is certain that the action is simply a chemical one, and is this:

What agents will disintegrate lime salts? We have

Carbonate,
Phosphate,
Fluate of Lime.

The greatest proportion is phosphate.

The answer to the question is familiar. *Acids.* And it is a true answer, so far as our knowledge goes. That it does not account for all the facts in the case is possibly due to our want of perfect knowledge of the conditions. It does not seem to account for peculiar methods of progression in caries. It does not seem to account for the fact that teeth seem to decay in pairs (though this is partly accounted for by the theory advanced in my previous paper;) that when the proximal surface of one molar is decayed we may usually look for a corresponding attack of the fellow tooth opposite. It does not account for the fact that decay seems sometimes to be spontaneously arrested; is self-limiting; is stationary; is resumed.

If, as Claude Bernard said in answer to a querist, we could see to the bottom of this question we could see to the bottom of all others—we should know all truth.

We assume then that acids are the cause of dental caries. From whence came these acids, and what is the method of their action? and what acids are they?

We may consider the saliva first: It is well to consider this as a *mixed* fluid, the product or secretion of the parotid, sub-maxillary, sub-lingual and mucous glands. The books say that saliva is an alkaline fluid, &c. But whatever may true of the saliva of typical man, we find that this *mixed* fluid is not alkaline usually, but acid. Out of 200 persons observed by one investigator seemingly in perfect health, 99 per cent. were found to have acid saliva except *just before meals.* These acids may be according to some writers, acetic, lactic, oxalic, uric, hydrochloric, nitric and sulphuric. It is certain that acetic, lactic, oxalic and hydrochloric have been found in the saliva; uric acid may be detected in the saliva in kidney troubles, and although nitric and sulphuric acids are not found, yet their presence is at least plausibly demonstrated. Sulphur is found in our food, as a proximate principle, and if the combination be dissolved it is set free, and in a nascent condition may readily form by combination with oxygen, sulphurous acid; or sulphurretted hydrogen, by union with hydrogen, and either of these I can conceive to be capable of being acted upon by other elements and sulphuric acid formed. The decomposition of nitrogenous particles of food may also by the liberation of nitrogen, allow of its combination with oxygen, and so the higher compounds of nitrogen and oxygen be formed.

Another and a prolific cause of dental caries may fairly be laid at the door of the medical practitioner. The administration of acid medicines is largely practised, and perhaps necessarily. But it is none the better for the teeth, that the administration is necessary. They are lost largely by this practice. It would be well to avoid whenever practicable the use of such drugs as tend to injure the enamel by combining with its lime-salts. The condition of the mouths of many sick people is bad enough for their teeth, with vitiated saliva and acid eructations from the stomach, without these additional troubles from without. And if it be true that the elimination of bromides and chlorides from the system by the salivary glande is a cause of caries, it would be well to know this and if possible guard against it.

My patients ask me, why do not my lower front teeth decay? It is a fact that as a rule these teeth are exempt from decay, as you are all well aware, but I have seen no theory which satisfactorily accounts for their escape. They are as freely bathed in saliva as are the other teeth, and while it is true they are in some cases protected by incrustations of salivary calculus, yet this calculus is porous and and permeable to acids, and must be saturated by the vitiated fluids of the mouth. The theory that they are protected from acid medicines by the lower lip and tongue is unsatisfactory also.

I called attention in the previous paper, to the peculiar location of occasional decay in the lower molars of children. The lingual faces of these teeth, never, or exceedingly seldom attacked by decay in adults, are seen to be extensively excavated in children. This, I should judge to be due to some peculiar conditions of the sub-lingual secretions or possibly of the mucous membrane of the sides of the tongue. Is it not possible that some pathological condition of the nervous system might affect the character of the secretions of certain glands, and is it possible further that some reflex action of the fifth pair might cause its peripheral fibres along the sides of the tongue to stimulate the secretion of an acid mucus?

I am more and more impelled to conclude, as I reflect on the conditions of caries of teeth, that it is ordinarily determined in good part by the secretions of the mouth, rather than by the decomposition of food retained between the teeth, and I conclude so for the reason that we see that the lower animals and inferior races of mankind are free from this affection, although under conditions which would, if the theory of food decomposition and its consequent acid action were true, be peculiarly liable to it, not possessing the means of tooth-cleansing of which we avail ourselves.

The question remains as to how far our vitiated tastes and abnormal habits influence the case. We see that the savage man is free from this affection, as are also the wild animals and even those which, though domesticated, preserve in tolerable distinctness their typical habits. How far does departure from a rigidly simple life tend to bring about this affection? Are we consuming proper food, and drinking proper fluids, and are we doing this at proper times and in proper quantities.? To ask these questions is to suggest the answer. Scalding hot viands smoke on our tables, and seething and boiling liquids furnish our drinks. We bolt food which the domesticated and almost humanized dog drops with a howl, and we swallow with zest fluids which would scald our fingers. We pass in an instant from the tea and coffee at nearlp 212° to ice water and frozen cream. We pile upon our food condiments which if applied to the external skin instantly produces irritation and soon becomes intolerable, and the stomach's walls and coats are supposed to be benefitted thereby. We are a nation of dyspeptics, a race who select—almost instinctively—food which does not require mastication and which is washed down without a particle of nature's lubricant, the saliva. We reject the natural forms of food, and insult natural tastes and appetites with leaven and yeast-powders, with diabolical messes of infernal cooking and of stomach-torturing mixtures. We reject the coarser foods, which natural man uses in large part, we use such food as does not require mastication, and as a consequence the teeth are not used; and there is strong reason for believing that the narrow jaw and contracted dental arch which is fast becoming typical of the American race, is due to a disuse of the jaws in childhood and a consequent lack of nervous flow to those parts. In a word, all our habits are those of untypical man, and the organs which are exposed with no vital protection suffer,—suffer in development, in imperfect organization, in lack of use, in speedy loss.

But while all this is only part of the truth, we stand utterly powerless to prevent these causes from producing their effect. Men and women, old and young, will not forbear even in the presence of pain, or in view of the inevitable and known penalty of transgression. Knowing, they heed not, and as the Holy Scriptures truly say "My people will not consider." "What has been, will be," I suppose, to the end of time, and possibly the evolutionist who has so learnedly argued the probability of an edentulous man—made edentulous from disuse of his teeth—is right, and in the millions of *eons* to come, the race will subsist on a pinch of food carried in the vest pocket and the stomach become atrophied or possibly dwindle into only a continuation in size of the œsophagus. This should be the outcome of evolution.

Meantime the several millions of generations suffer, and *must* find relief. And so a profession has arisen—that of Dentistry.

I must not repeat what we all know so well, that no operation on the human organism is so uniformly successful as that of tooth-filling, nor need I inform you of what you are well aware, that thousands of teeth are sacrificed by a want of knowledge of this fact. Nor need I tell you that where these organs are lost and the last resort of extraction comes, that artificial substitutes are made which for perfection of imitation of nature's work are beyond comparison with anything artificial, unless it be the making of artificial flowers; that the sunken lips, the aged mouth, the changed features are restored, and rejuvination given to the features "made after the divine image." Nor need I tell you that these substitutes in addition to their wondrous naturalness of appearance, are so useful in mastication that dyspeptics are cured and appetites improved by their proper insertion.

All these are familiar facts.

It remains to give the sum of the results of the studies of the dental profession as to dental hygiene and prophylacsis; of the best methods of filling teeth, (not of course describing these methods,) of dental therapeutics and dental prosthesis. But as I am not writing a book, but only a brief paper, and as your time has already been encroached upon, and as I fear to be likened to the terrible dental engine which remorsely bores, I stop.

